# Hémangiome vertébral neuro-agressif de révélation tardive: cas clinique A clinical case of late-onset aggressive vertebral hemangioma with neurological signs

**DOI:** 10.11604/pamj.2020.37.218.24313

**Published:** 2020-11-05

**Authors:** Komi Ignéza Agbotsou, Damelan Kombate, Amégninou Mawuko Yao Adigo, Kossivi Apétsè, Albert Beschet, Victor Chan

**Affiliations:** 1 Service de Neurologie du Centre Hospitalier de Valence, Valence, France,; 2 Service de Neurologie du Centre Hospitalier Universitaire Campus, Lomé, Togo,; 3 Service de Radiologie et Imagerie Médicale du Centre Hospitalier Régional de Bè, Lomé, Togo

**Keywords:** Hémangiome, neuro-agressif, vertébral, Hemangioma, aggressive with neurological signs, vertebral

## Abstract

Résumé

Dans la majorité des cas asymptomatiques, les hémangiomes vertébraux peuvent être, dans de rares cas, symptomatiques avec des manifestations cliniques purement neurologiques. S´ils sont fréquemment observés chez un sujet adulte jeune, ils peuvent exceptionnellement être observés chez un sujet âgé. Nous rapportons un cas d´hémangiome vertébral neuro-agressif de révélation tardive traité par une chirurgie décompressive, une sclérothérapie, une cimentoplastie et suivi d´une évolution favorable.

English abstract

In the majority of asymptomatic cases, vertebral hemangiomas can be, in rare cases, symptomatic with purely neurological clinical manifestations. They commonly occur in young adults, exceptionally in elderly subjects. We here report a case of late onset aggressive vertebral hemangioma with neurological signs treated with decompressive surgery, sclerotherapy and cementoplasty, with favorable outcome.

## Introduction

Les hémangiomes vertébraux sont des tumeurs bénignes, d´origine malformative, constituées de vaisseaux sanguins néoformés de structure normale, sans shunt artérioveineux [1]. Ce sont des lésions fréquentes [2] qui, dans l´immense majorité des cas, demeurent quiescentes et asymptomatiques [1]. Les formes évolutives responsables d´une compression médullaire sont beaucoup plus rares [3]; elles relèvent d´une prise en charge neurochirurgicale après une imagerie médullaire associée à une biopsie s´il existe un doute diagnostic.

## Patient et observation

Il s´agit d´une patiente de 72 ans, ayant un antécédent d´insuffisance antéhypophysaire suivie et sous traitement hydrocortisone et levothyrox, porteuse d´un virus de l´hépatite C traité par Harvoni trois mois avant son hospitalisation, qui a été admise pour des troubles de la marche et de l´équilibre avec des chutes à répétition et des troubles sensitifs à type de brûlure remontant jusqu´au niveau de l´ombilic accompagnés de troubles sphinctériens à type de dysurie et de miction impérieuse, le tout d´aggravation progressive depuis 2 mois avant son hospitalisation. A l´admission, l´examen physique a noté une para parésie avec la force motrice à 4/5 des 2 côtés à la manœuvre de Barré, les réflexes ostéotendineux rotuliens étaient vifs mais non diffusés des 2 côtés avec une trépidation épileptoïde discrète, la tenue sur la pointe et le talon des pieds était impossible de même que la marche en funambule, un signe de Babinski bilatéral et une hypoesthésie avec un niveau en T7. L´examen du rachis a noté une douleur modérée en T7, T9, T10 sans déformation du rachis cervico-dorso-lombaire.

**Figure 1:**
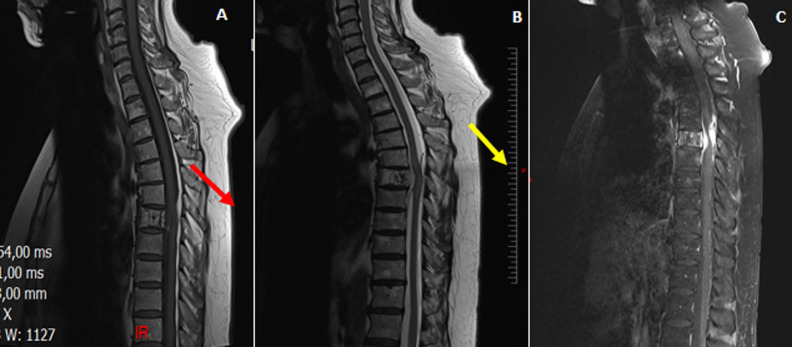
lésion vertébrale en isosignal T1; A) et isosignal T2; B) prenant le contrast de façon intense; C) avec une expansion épidurale ; épidurite T7 compressive sur lésion vertébrale suspecte avec deux autres lésions en T3 T9 sans épidurite

**Figure 2:**
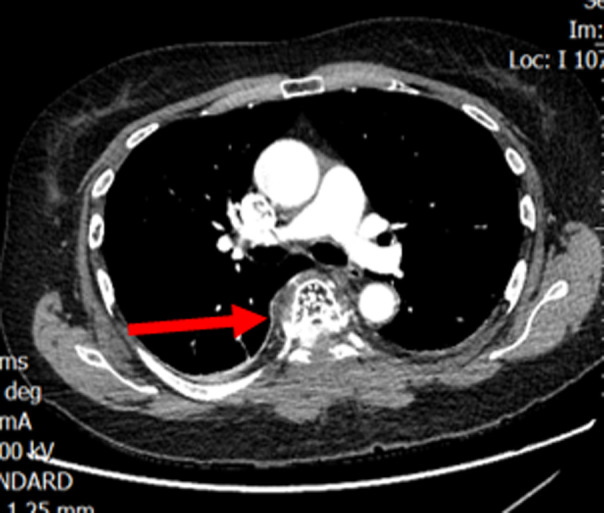
scanner thoraco-abdomino-pelvien avec injection de produit de contraste iodé: coupe axiale passant par T7, aspect piqueté épais d´os dense (aspect en têtes d´épingles) de l´hémangiome vertébrale en T7 avec extension postérieure intra-canalaire et à l´arc postérieur

L´examen des membres supérieurs était normal. Il a été conclu un syndrome de compression médullaire lente de niveau T7. Elle a bénéficié d´une IRM médullaire qui a montré une épidurite T7 compressive sur lésion vertébrale suspecte avec deux autres lésions en T3 T9 sans épidurite (Figure 1). Un scanner thoraco-abdomino-pelvien (Figure 2) réalisé à la recherche d´une néoplasie n´avait pas montré de lésion suspecte au niveau de l´étage thoraco-abdomino-pelvien. Devant la présence des signes cliniques et radiologiques de compression médullaire en T7, dans un premier temps, elle a été prise en neurochirurgicalement pour une laminectomie dorsale en regard de T7 et une décompression médullaire sans ostéosynthèse ni exérèse de la tumeur avec des suites opératoires simples. L´examen anatonomo-cyto-pathologie réalisée, devant la suspicion de signe de malignité, sur la pièce de biopsie lors du temps opératoire, confirme une tumeur épidurale et fragments vertébraux d´un hémangiome avec une absence de signe histologique de malignité ou de granulome caséeuse giganto-cellulaire.

**Figure 3:**
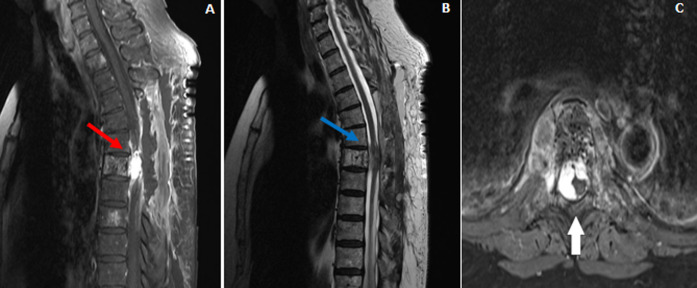
IRM médullaire post-laminectomie décompressive; persistance de l´hémangiome agressif de T7 avec extension postérieure épidurale (A,B,C)

Une Imagerie par Résonance Magnétique (IRM) médullaire de contrôle post-laminectomie décompressive (Figure 3) a été réalisée qui montre une persistance de l´hémangiome agressif de T7 avec extension postérieure épidurale. Sur le plan biologique, on note une absence du syndrome inflammatoire biologique avec une C Réactive Protéine (CRP) à 3mg/l et les leucocytes à 8700 éléments/mm^3^. Le bilan d´auto-immunité était négatif. Les sérologies VIH, Lyme, syphilis étaient négatives. Il n´avait pas de trouble ionique et la fonction rénale était normale. Elle a bénéficié ensuite d´une sclérothérapie et d´une cimentoplastie sans complication notable dans les suites. L´évolution sur le plan clinique a été favorable avec une correction des troubles moteurs des membres inférieurs (force motrice à 5/5 aux membres inférieurs) avec une équilibre bonne en station debout, une tenue sur la pointe des pieds et sur les talons possibles et une amélioration des troubles sphinctériens avec la régression de la dysurie et la miction impérieuse.

## Discussion

La forme asymptomatique des hémangiomes vertébraux est très fréquente (10% des autopsies) et représente la plus fréquente des tumeurs bénignes vertébrales [1]. Ils sont découverts à l´occasion d´un examen réalisé à titre systématique ou suite à un traumatisme. La forme neuro-agressive est très rare et se retrouve dans 1% des cas [3]. Comme chez notre patiente, La localisation dorsale des hémangiomes neuro-agressifs est la plus fréquente, habituellement entre la 3^e^et la 9^e^vertèbre dorsale [4]. Elle se voit chez l´adulte jeune entre 40 ans et 60ans avec une légère prédominance féminine, deux femmes pour un homme [4]. La forme tardive (72 ans) que nous rapportons, à notre connaissance, a été très rarement décrite dans la littérature.

La symptomatologie classique de la forme neuro-agressive des hémangiomes vertébraux est le syndrome de compression médullaire et/ou radiculaire d´installation progressive [5]. Chez notre patiente, le syndrome de compression médullaire a été le mode révélateur avec des troubles moteurs aux membres inférieurs et un trouble sensitif à type de brûlure et un trouble urinaire. L´extension progressive de l´hémangiome dans le canal rachidien est le plus souvent responsable de cette compression; la forme péjorative est celle qui se présente comme une para-parésie ou tétraplégie aiguë suite à un tassement vertébral. L´IRM occupe une place incontournable dans le diagnostic par le fait qu´elle permet une analyse multiplanaire directe et représente un pouvoir de caractérisation tissulaire [6].

L´aspect habituel est caractérisé par un hyper signal osseux sur les séquences pondérées en T1 et T2. La perte de l´hyper signal sur les séquences spin écho T1 est un des critères d´agressivité de l´angiome [6, 7]. Elle trouve son intérêt majeur dans le bilan d´extension au niveau épidural avec une bonne approche du retentissement sur les structures nerveuses médullaires et radiculaires. Dans notre cas, la biopsie vertébrale est réalisée, en plus de l´IRM, devant le doute diagnostic vu la localisation multi-vertébrale et le caractère suspect de la lésion. En effet, s´il y a un doute diagnostique, une biopsie du corps vertébral ou de l´arc postérieur s´impose [8]. Les caractères d´agressivité de l´hémangiome sont, une localisation entre la 3^e^ vertèbre dorsale et la 10^e^vertèbre dorsale, l´atteinte de l´ensemble du corps vertébral, l´extension à l´arc postérieur, l´aspect soufflé, aminci et discontinu des corticales, la présence de plages lytiques étendues au sein de l´angiome, l´extension dans les parties molles para-vertébrales ou intra-canalaire et à l´IRM, un signal tissulaire avec une prise de contraste intense [6].

La compression médullaire impose de toute façon un geste de décompression, précédé si possible par une embolisation. Il s´agit le plus souvent d´une laminectomie si la compression est circonférentielle ou postérieure. Chez notre patiente, l´indication de la cimentoplastie en plus de la sclérothérapie et de la chirurgie décompressive a été posée du fait de la présence du syndrome rachidien douloureux. En effet, le syndrome vertébral douloureux est l´indication principale de la cimentoplastie [9]. La revue de littérature indique qu´en présence de signes neurologiques, la vertébroplastie peut être associée à une laminectomie de décompression [9]. En cas de laminectomie isolée, le taux de rechute est important, variant de 27 à 38% [10].

## Conclusion

Les hémangiomes vertébraux agressifs représentent une entité très rare et exceptionnelle chez le sujet âgé. Ils se manifestent le plus souvent par des signes de compression médullaire d´installation progressive, le risque majeur est sa présentation d´emblée par une paraplégie ou une tétraplégie nécessitant une chirurgie décompressive urgente.
